# New strategies for drug discovery: activation of silent or weakly expressed microbial gene clusters

**DOI:** 10.1007/s00253-012-4551-9

**Published:** 2012-11-11

**Authors:** Kozo Ochi, Takeshi Hosaka

**Affiliations:** 1 Department of Life Science, Hiroshima Institute of Technology, Miyake 2-1-1, Saeki-ku, Hiroshima, 731-5193 Japan; 2 Department of Bioscience and Biotechnology, Faculty of Agriculture, Shinshu University, 8304 Minamiminowa, Nagano, 399-4598 Japan

**Keywords:** Silent gene activation, Strain improvement, Ribosome engineering, Rare earth elements

## Abstract

Genome sequencing of *Streptomyces*, myxobacteria, and fungi showed that although each strain contains genes that encode the enzymes to synthesize a plethora of potential secondary metabolites, only a fraction are expressed during fermentation. Interest has therefore grown in the activation of these cryptic pathways. We review current progress on this topic, describing concepts for activating silent genes, utilization of “natural” mutant-type RNA polymerases and rare earth elements, and the applicability of ribosome engineering to myxobacteria and fungi, the microbial groups known as excellent searching sources, as well as actinomycetes, for secondary metabolites.

## Introduction

Applied microbiology seeks, for example, to identify the latent activities of microorganisms and to enhance these activities at the industrial level by strain improvement. Current methods of strain improvement, ranging from classical random approaches to metabolic engineering, however, are either costly or labor-intensive (Santos and Stephanopoulos 2008). Moreover, after 1970, the rate of discovery of useful compounds declined progressively, despite the constant need for new and improved drugs to combat emerging and reemerging infectious diseases and cancer. Recent advances in DNA sequencing technologies have enabled multiple genomes to be sequenced rapidly and inexpensively. *Streptomyces* genome sequencing showed that each strain contains genes that encode the enzymes to synthesize 20 or more potential secondary metabolites (Bentley et al. 2002; Ikeda et al. 2003; Ohnishi et al. 2008), only a fraction of which are expressed during fermentation. These cryptic biosynthetic pathways may produce many novel bioactive compounds with the potential to rejuvenate stalled drug discovery pipelines. Methods to activate these silent biosynthetic pathways are thus of major interest. The key issue for the success of this approach is to find ways to induce or enhance the expression of cryptic or poorly expressed pathways to provide material for structure elucidation and biological testing. Thus, strategies for rapid strain improvement will likely shift from the improved expression of well-known, highly productive, secondary metabolites of fermentation to the expression of novel and often cryptic secondary metabolite pathways (Baltz 2011). This approach may solve the early stage discovery problems of: (a) inducing some level of expression of cryptic biosynthetic gene clusters [waking the sleeping genes] and (b) rapidly increasing product yields to obtain enough material to characterize chemically and biologically [early stage yield enhancement].

The notion of “ribosome engineering” originally came from the finding, that a *Streptomyces lividans* strain with an altered ribosomal S12 protein that confers streptomycin resistance produced abundant quantities of the blue-pigmented antibiotic actinorhodin, although *S*. *lividans* normally does not produce antibiotics due to the dormancy of the antibiotic biosynthesis genes (Shima et al. 1996). On the other hand, the bacterial alarmone ppGpp, produced on the ribosome, was found to bind to RNA polymerase (RNAP) (Artsimovitch et al. 2004), eventually initiating the production of antibiotics (Bibb 2005; Ochi 2007). This suggested that RNAP modification, by introducing a rifampicin resistance mutation, may mimic the ppGpp-bound form, activating the expression of biosynthetic gene clusters (Lai et al. 2002; Xu et al. 2002). Consequently, we have developed a method, termed ribosome engineering, to activate or enhance the production of secondary metabolites by targeting ribosomal protein S12, as well as other ribosomal proteins and translation factors, or RNAP, hypothesizing that bacterial gene expression may be increased dramatically by altering transcription and translation pathways.

Ribosome engineering is characterized by its applicability to both strain improvement and silent gene activation to identify novel secondary metabolites. The fundamental mechanism by which ribosome engineering affects antibiotic production has been summarized in earlier reviews (Ochi et al. 2004; Ochi 2007), as has the outline of this technology (Baltz 2011; Chiang et al. 2011; Olano et al. 2008; Xie et al. 2009). Therefore, the present review highlights recent advances on this topic.

## Impact on strain improvement

Since many antibiotics, such as streptomycin, target the ribosome, ribosome mutants that confer antibiotic resistance may be obtained by simply selecting mutants on drug-containing plates, although some fraction of the mutants may be the ones affected in membrane permeability. Similarly, RNAP mutants may be obtained by growing bacteria on plates containing rifampicin that targets RNAP. This feasibility has yielded many successful examples of ribosome engineering, including the enhanced production of secondary metabolites and enzymes, as well as enhanced tolerance to toxic compounds such as 4-hydroxybenzoate (Table 1). Ribosome engineering was effective in enhancing the yield of secondary metabolites in a wide range of structural classes, including polyketides, macrolides, aminoglycosides, and nucleosides. Importantly, the K88E and K88R mutations in *rpsL* (polypeptide amino acid numbering according to *Streptomyces coelicolor*), which encodes the ribosomal protein S12, and the H437Y and H437R in *rpoB*, which encodes the RNAP β subunit, were often effective (Table 1). In all these mutant strains of *S*. *coelicolor*, overproduction of actinorhodin correlated with higher expression of *act II*-ORF4, a pathway-specific positive regulator of actinorhodin synthesis. Combinations of these drug-resistance mutations further enhanced bacterial productivity (Tamehiro et al. 2003; Tanaka et al. 2009a). For example, the introduction of eight different mutations enhanced actinorhodin production by *S*. *coelicolor* 280-fold (Wang et al. 2008) and the introduction of three mutations enhanced the production of the enzyme cycloisomaltooligosaccharide glucanotransferase by *Paenibacillus agaridevorans* 1,000-fold (Tanaka and Ochi, manuscript in preparation). Mutations in *rpsL* enhanced expression of the *frr* gene, which encodes ribosome recycling factor (Hosaka et al. 2006), and overexpression of *frr* in *Streptomyces avermitilis* increased avermectin production, even in an industrial strain (Li et al. 2010). Overexpression of *frr* may be a general method of boosting translation during the stationary phase, leading to reinforcement of secondary metabolism. The *rif* mutation S444F increased erythromycin production by *Saccharopolyspora erythraea* fourfold and metabolic changes induced by this mutation were analyzed in detail using DNA microarrays (Carata et al. 2009).

**Table 1:** Improvement of antibiotic/enzyme production and cell’s physiology by subjecting to ribosome engineering

Strain	Antibiotic/enzyme	Mutation	Comment	Reference
Actinomycetes				
*S*. *coelicolor*	Actinorhodin	*rpsL*(K88E or K88R)	Using *relC* mutant	Ochi et al. (1997)
*S*. *coelicolor*	Actinorhodin	*rpsL*(K88E)	Streptomycin resistance	Hesketh and Ochi (1997)
*S*. *coelicolor*	Actinorhodin	*rpsL*(P91S)	Paromomycin resistance	Okamoto-Hosoya et al. (2000)
*S*. *coelicolor*	Actinorhodin	*rpsL*(K88E) gen *rpoB*(H437Y)	Triple mutation	Hu and Ochi (2001)
*S*. *coelicolor*	Actinorhodin	*rpoB*(R440H or Q424L)	Using *relA* and *relC* mutants	Xu et al. (2002)
*S*. *coelicolor*	Actinorhodin	*rpsL*(K88E)	Streptomycin resistance	Hosaka et al. (2006)
*S*. *coelicolor*	Actinorhodin	*rsmG*	Function-loss mutation	Nishimura et al. (2007)
*S*. *coelicolor*	Actinorhodin	*rpsL* gen *rpoB* par gnt fus tsp lin	Octuple mutation	Wang et al. (2008)
*S*. *coelicolor*	Actinorhodin	*rsmG*	Function-loss mutation	Tanaka et al. (2009a)
*S*. *coelicolor*	Actinorhodin	*rsmG rspL*(R86P or K88E)	Double mutation	Tanaka et al. (2009a)
*S*. *coelicolor*	Actinorhodin	*rpsL*(K88E)	Streptomycin resistance	Tanaka et al. (2009a)
*S*. *coelicolor*	Actinorhodin	*rpsL*(K88E GI92)	Double mutation with paromomycin resistance	Wang et al. (2009a)
*S*. *coelicolor*	Chloramphenicol	*rpsL*(K88E) *rpoB*(S433L)	Double mutation, heterologous expression	Gomez-Escribano and Bibb (2011)
*S*. *coelicolor*	Actinorhodin	ery	Erythromycin resistance	Imai et al. (2012)
*S*. *lividans*	Actinorhodin	*rpsL*(K88E)	*S*. *lividans* TK24 with str	Shima et al. (1996)
*S*. *lividans*	Actinorhodin	*rpoB*(S433L or S433P)	Rifampicin resistance	Hu et al. (2002)
*S*. *lividans*	Actinorhodin	*rpoB*(R440C)	Using *relC* mutant	Lai et al. (2002)
*S*. *lividans*	Actinorhodin	*rpsL*(L90K or R94G)	Site-directed mutagenesis	Okamoto-Hosoya et al. (2003)
*S*. *lividans*	Actinorhodin	*rsmG*	Function-loss mutation	Nishimura et al. (2007)
*S*. *lividans*	Actinorhodin	cap	Capreomycin resistance	Zhang et al. (2008)
*S*. *lividans*	Actinorhodin	*rpoB*(H426N, S431N, F445M, S474Y, M581D)	“Natural” mutant-type *rpoB*	Tala et al. (2009)
*S*. *lividans*	Actinorhodin	ery	Erythromycin resistance	Imai et al. (2012)
*S*. *antibioticus*	Actinomycin	str	Streptomycin resistance	Hosoya et al. (1998)
*S*. *antibioticus*	Actinomycin	*rsmG*	Function-loss mutation	Tanaka et al. (2009a)
*S*. *antibioticus*	Actinomycin	*rpsL*(K88E)	Streptomycin resistance	Tanaka et al. (2009a)
*S*. *antibioticus*	Actinomycin	*rsmG rspL*(K88E or K88R)	Double mutation	Tanaka et al. (2009a)
*S*. *parvulus*	Actinomycin	*rpsL*(K88R)	Streptomycin resistance	Tanaka et al. (2009a)
*S*. *parvulus*	Actinomycin	*rsmG*	Function-loss mutation	Tanaka et al. (2009a)
*S*. *parvulus*	Actinomycin	ery	Erythromycin resistance	Imai et al. (2012)
*S*. *griseus*	Streptomycin	*rsmG*	Function-loss mutation	Tanaka et al. (2009b)
*S*. *griseus*	Streptomycin	ery	Erythromycin resistance	Imai et al. (2012)
*S*. *erythraea*	Erythromycin	*rpsL*(K43N)	Streptomycin resistance	Tanaka et al. (2009a)
*S*. *erythraea*	Erythromycin	*rpoB*(S444F)	Rifampicin resistance	Carata et al. (2009)
*S*. *albus*	Salinomycin	*rpsL*(K88R) gen rif	Triple mutation in an industrial strain	Tamehiro et al. (2003)
*S*. *avermitilis*	Oligomycin	*rpsL*(K43M or K88E)	Streptomycin resistance	Tanaka et al. (2009a)
*S*. *bingchenggensis*	Milbemycin	str	Streptomycin resistance	Wang et al. (2009b)
*S*. *chattanoogensis*	Fredericamycin	str	Streptomycin resistance	Hosoya et al. (1998)
*S*. *fradiae*	A54145	*rpsL*(K88R)	Streptomycin resistance	Alexander et al. (2010)
*S*. *incarnatus*	Sinefungin	*rpoB*(D447G)	Rifampicin resistance	Fukuda et al. (2010)
*S*. *lavendulae*	Formycin	str	Streptomycin resistance	Hosoya et al. (1998)
*S*. *mauvecolor*	Piperidamycin	*rpoB*(H437L), gen, or *rpoB rpsL*(K88R)	Single or double mutation	Hosaka et al. (2009)
*S*. *roseosporus*	A21978C	*rpsL*(K43N)	Streptomycin resistance	Wang et al. (2012)
*S*. *viridochromogenes*	Xylanase	*rpsL*(K88R)	Streptomycin resistance	Liu et al. (2012)
*A*. *orientalis*	Norvancomycin	str rif	Double mutation	Wang et al. (2006)
*P*. *rosea*	GE2270	str gen rif	Triple mutation in an industrial strain	Beltrametti et al. (2006)
*Nonomuraea* sp.	Glycopeptide A40926	*rpoB*(H426N, S431N, F445M, S474Y, M581D)	"Natural" mutant-type *rpoB*	Vigliotta et al. (2005)
*Streptomyces* sp.	Antibiotics	str	Streptomycin resistance	Hai et al. (2011)
Actinomycete	Antitumor activity	str	Streptomycin resistance	Han et al. (2009)
Actinomycete	Antitumor activity	str	Streptomycin resistance	Sun et al. (2010)
Actinomycete	Antitumor activity	str	Streptomycin resistance	Han et al. (2010)
Eubacteria				
*E*. *coli*	Cell-free protein synthesis	*rpsL*(K87E)	Streptomycin resistance	Hosaka et al. (2004)
*E*. *coli*	Cell-free protein synthesis	*rpsL*(K42T)	Streptomycin resistance	Chumpolkulwong et al. (2004)
*E*. *coli*	Replication progression	*rpoB*(H447R)	Rifampicin resistance	Baharoglu et al. (2010)
*E*. *coli*	Violacein	lin kan	Heterologous gene expression	Ahmetagic and Pemberton (2011)
*B*. *subtilis*	Neotrehalosadiamine	*rpoB*(S487L)	Rifampicin resistance	Inaoka et al. (2004)
*B*. *subtilis*	α-Amylase	*rpsL*(K56R)	Streptomycin resistance	Kurosawa et al. (2006)
*B*. *subtilis*	Sporulation, germination, competence	*rpoB*(H482R, H482Y)	Rifampicin resistance	Maughan et al. (2004)
*B*. *subtilis*	Sporulation and tolerance	*rpoB*	Rifampicin resistance	Moeller et al. (2012)
*B*. *cereus*	FR900493	str	Streptomycin resistance	Hosoya et al. (1998)
*C*. *acetobutylicum*	Aceton-Butanol	str	Streptomycin resistance	Gao et al. (2012)
*D*. *radiodurans*	Radiation resistance	*rpoB*(L420R)	Rifampicin resistance	Hua et al. (2011)
*P*. *luminescens*	Nematicidal activity	*rpoB*(P564L)	Rifampicin resistance	Qiu et al. (2012)
*P*. *putida*	4-HBA resistance	*rpsL*(K88E) or *rpoB*(S517P)	Resistance to toxic compounds	Hosokawa et al. (2002)
*P*. *pyrrocinia*	Pyrrolnitrin	str	Streptomycin resistance	Hosoya et al. (1998)
Fungi				
*P*. *purprogenum*	Antitumor activity	gen	Gentamicin resistance	Chai et al. (2012)

The genetic symbols, cap, ery, fus, gen, gnt, kan, lin, par, rif, str, and tsp indicate resistance to capreomycin, erythromycin, fusidic acid, gentamicin, geneticin, kanamycin, lincomycin, paromomycin, rifampicin, streptomycin, and thiostrepton, respectively. *rpsL* and *rpoB* indicate the genes coding for the ribosomal protein S12 and the RNA polymerase β-subunit, respectively

Construction of an amenable host organism for heterologous gene expression is a current goal for enhancing yield and activating cryptic gene clusters (Baltz 2010; Komatsu et al. 2010). Introduction of the *rpsL* K88R mutation into *Streptomyces fradiae* resulted in the efficient heterologous expression of secondary metabolite genes (Alexander et al. 2010). Moreover, introduction of the *rpsL* K88E and *rpoB* S433L mutations into *S*. *coelicolor* enhanced the production of chloramphenicol and congocidine 40- and 30-fold, respectively (Gomez-Escribano and Bibb 2011), and mutations conferring resistance to lincomycin and kanamycin enhanced violacein production in *Escherichia coli* 41-fold (Ahmetagic and Pemberton 2011). Although the present review does not focus on the host organism system for heterologous expression of secondary metabolite gene clusters, the most updated review can be seen elsewhere (Komatsu et al. 2013).

The *rsmG* gene encodes a 16S rRNA methyltransferase, which methylates position G527 of the 530 loop of 16S rRNA (Okamoto et al. 2007). Mutations of this gene in *Streptomyces* strains confer low-level resistance to streptomycin and cause preferential overexpression of the *metK* gene (at least in *S*. *coelicolor*), which encodes the enzyme *S*-adenosylmethionine (SAM) synthetase, eventually leading to an increase in intracellular SAM level (Nishimura et al. 2007). This increase, together with enhanced protein synthesis during the late growth phase, results in overproduction of antibiotic (Kim et al. 2003; Okamoto et al. 2003). Addition of SAM to the medium or propagation of *metK* with multicopy plasmids also results in antibiotic overproduction by various *Streptomyces* spp. and other actinomycetes (Huh et al. 2004; Maharjan et al. 2008, 2012; Oh et al. 2010; Paudel et al. 2011; Saito et al. 2003; Shin et al. 2007; Sun et al. 2012; Wang et al. 2007; Zhao et al. 2006, 2010). Spontaneous *rsmG* mutations arise at a high frequency (10^−4^–10^−6^) (Nishimura et al. 2007; Okamoto et al. 2007). *rsmG* mutation (and also *ksgA* mutation [Ochi et al. 2009]) harbors several mysterious features (Nodwell 2007) and is effective for not only enhanced antibiotic production but also activation of cryptic secondary metabolite biosynthetic genes (Tanaka et al. 2009b). When constructing strains combining low-level (*rsmG*) and high-level (*rpsL*) streptomycin resistance mutations, the mutations should be introduced in that order (Tanaka et al. 2009a).

## Impact on silent gene activation

Promising approaches for the activation of cryptic biosynthetic gene clusters in *Streptomyces* species include ribosome engineering; the addition of *N*-acetylglucosamine to the medium or deletion of the *dasR* gene, which encodes an *N*-acetylglucosamine-responsive regulatory protein, the constitutive overexpression of a pathway-specific large ATP-binding LuxR-type (LAL) regulatory gene, metabolic remodeling, and cell-to-cell interaction. These new approaches may enable the identification of novel and/or poorly understood antibiotics, such as piperidamycin and stambomycin, and are all characterized by applicability to a wide range of actinomycetes and potential scalability to high throughput.

### Ribosome engineering

**Fig. 1:**
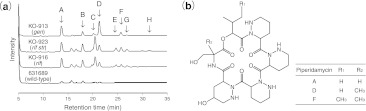
Detection of antibacterial compounds produced by drug-resistant mutants of *S. mauvecolor* 631689. **a** HPLC profiles of culture extracts. The *arrows* (*A*–*H*) indicate the bioactive fractions. **b** The chemical structures of the antibacterial compounds isolated

Ribosome engineering is effective for activation of silent genes. For example, among the 1,068 actinomycetes isolated from soil, a fraction of the *Streptomyces* isolates and most of the non-*Streptomyces* isolates were found to be nonproducers of antibiotics, with 43 and 6 %, respectively, of these nonproducing strains acquiring the ability to synthesize antibacterials against *Staphylococcus aureus* after a selection step that generated spontaneous *rpsL* or *rpoB* mutations (Hosaka et al. 2009). Assessment of *Streptomyces mauvecolor* 631689, a strain that produced no antibacterial activity in any medium tested, demonstrated that two *rpoB* mutants (H437D or H437L), a double mutant of *rpoB* (H437L) and *rpsL* (K88R), and a gentamicin-resistant (GenR) mutant produced a family of antibiotics, the piperidamycins (Fig. 1). The activation of silent genes by the *rpoB* H437D or H437L mutations was attributed, at least in part, to the increased affinity of mutant RNAP for the silent gene promoters (Hosaka et al. 2009).

Species of *Bacillus* produce a variety of commercially important metabolites and extracellular enzymes. The introduction of the *rpoB* mutation S487L into a *Bacillus subtilis* strain resulted in cells that overproduced an aminosugar antibiotic, 3,3′-neotrehalosadiamine (NTD), the production of which is dormant in the wild-type strain (Inaoka et al. 2004). Perhaps, unlike the wild-type RNAP, the mutant RNAP efficiently recognized the *σ* ^A^-dependent promoters, resulting in the dramatic activation of the NTD biosynthesis pathway. Although most of the work was performed using *S*. *lividans*, we have now demonstrated that *rpoB* mutations are widely effective in activating silent and poorly expressed secondary metabolite-biosynthetic gene clusters at the transcriptional level in *Streptomyces griseus* (up to 70-fold activation), *S*. *coelicolor* (up to eightfold activation), and *S. erythraea* (up to sevenfold activation; Tanaka and Ochi, manuscript in preparation). Notably, the activation of silent gene clusters by *rpoB* mutations was medium-dependent, with each *rpoB* mutation exerting differential effects on the activation of each silent gene cluster. These findings suggest that strains containing *rpoB* mutations (e.g., H437Y, H437R) should be grown in different media to assess the full spectrum of silent gene activation.

The recent observation, that several actinomycetes possess two *rpoB* genes (Vigliotta et al. 2005), has suggested a new strategy of activating silent gene expression in bacteria. Two *rpoB* paralogs, *rpoB*(*S*) and *rpoB*(*R*), provide *Nonomuraea* sp. strain 39727 with two functionally distinct and developmentally regulated RNAPs. The product of *rpoB*(*R*), the expression of which increases after transition to stationary phase, is characterized by five amino acid substitutions (H426N, S431N, F445M, S474Y, and M581D) located within or close to the rifampicin resistance cluster. The expression of *rpoB*(*R*) was found to markedly activate antibiotic biosynthesis, with the *rpoB*(*R*)-specific H426N mutation found essential in activating secondary metabolism (Tala et al. 2009). Additional *rif* cluster-associated *rpoB*(*R*)-specific missense mutations likely interact functionally with the H426N mutation, leading to the marked effect of *rpoB*(*R*). Mutant type, or duplicated, *rpoB* often exists in nature, with *rpoB* gene polymorphisms detected in five of 75 inherently rifampicin-resistant actinomycetes isolated from nature, although these polymorphisms were preferentially distributed in the so-called rare actinomycetes, not in *Streptomyces* spp. Notably, all but one of these rifampicin-resistant rare actinomycete isolates obtained to date were able to produce antibiotics (Tala et al. 2009).

From a practical viewpoint, these findings suggest the intriguing possibility of using *rpoB*(*R*)-based technology to improve strains and to search for novel bioactive molecules by activating silent genes. This technology should have greater potential than the simple *rif* selection currently used to improve the production of secondary metabolites, as the introduction of *rpoB*(*R*) enhanced antibiotic production eightfold when compared with the introduction of the H426Y mutation (Tala et al. 2009). It is also of interest to examine whether various *rpoB*(*R*) forms found in nature are more capable of activating silent bacterial genes than *Nonomuraea rpoB*(*R*). Thus, understanding the status of natural *rpoB*(*R*) and utilizing it for cryptic gene activation may provide new horizons for medical and industrial microbiology.

### *dasR–N*-acetylglucosamine system

DasR is a global regulator of antibiotic biosynthesis, which links nutrient stress to antibiotic production by *Streptomyces* (van Wezel and McDowall 2011). *N*-acetylglucosamine (GlcNAc), a major component of the cell walls of fungi, is the second most abundant polysaccharide in nature. A high concentration of GlcNAc, perhaps mimicking its accumulation after autolytic degradation of the vegetative mycelium, may be a major checkpoint for the onset of secondary metabolism (Rigali et al. 2008). The response is transmitted to antibiotic pathway-specific activators through the pleiotropic transcriptional repressor DasR, suggesting a new strategy for activating pathways of secondary metabolite biosynthesis. In *S*. *coelicolor*, GlcNAc (∼10 mM) blocks development and antibiotic production under rich growth conditions, but triggers antibiotic production and sporulation under poor nutritional conditions (i.e., minimal medium), suggesting that the effect of GlcNAc depends on the specific culture conditions. GlcNAc also stimulated antibiotic production by other *Streptomyces* spp., including *Streptomyces clavuligerus*, *Streptomyces collonus*, *S*. *griseus*, *Streptomyces hygroscopicus*, and *Streptomyces venezuelae* (Rigali et al. 2008).

The GlcNAc regulon is controlled by the GntR regulator DasR, the DNA-binding activity of which is inhibited by glucosamine-6-phosphate. Introduction of mutations into *dasR* effectively enhanced antibiotic productivity. For example, the *S*. *coelicolor dasR* mutant BAP29 showed three- to fourfold enhanced and accelerated production of pigmented antibiotics (Rigali et al. 2008). The position of the DasR binding site in the *act II*-ORF4 promoter indicates that DasR represses transcription of this gene. Interestingly, the *dasR* mutant BAP29 was found to be awakened for cryptic gene clusters encoding a hypothetical antibiotic made by a type I modular polyketide synthase (SCO6273-6288), showing that this method is applicable for activating silent gene clusters (Rigali et al. 2008).

### LAL regulatory system

Constitutive overexpression of a putative pathway-specific LAL regulator was shown to successfully induce the expression of the silent type I modular polyketide synthase (PKS) gene cluster in *Streptomyces ambofaciens* 23877, enabling the identification of a unique structural class of polyketides with promising antitumor activity (Laureti et al. 2011). *S*. *ambofaciens* 23877 produces two antibiotics, the macrolide spiramycin and the pyrrole-amide congocidine. Sequence analysis of its genome showed that *S*. *ambofaciens* 23877 contains several secondary metabolite biosynthetic gene clusters, including a giant type I modular PKS gene cluster. This cluster is composed of 25 genes, nine of which encode PKSs, and spans almost 150 kb, making it one of the largest polyketide biosynthetic gene clusters described to date. The metabolic product(s) of this gene cluster have not been determined and transcriptional analyses showed that this cluster is not expressed under laboratory growth conditions. The constitutive expression of a regulatory gene within this cluster, encoding a protein that is similar to the LAL family of proteins, triggered the expression of the biosynthetic genes. This led to the identification of four 51-membered glycosylated macrolides, named stambomycins A–D, as metabolic products of the gene cluster (Laureti et al. 2011). Database searches have identified genes that encode LAL regulators within numerous cryptic biosynthetic gene clusters in actinomycete genomes, for which metabolic product(s) remain to be discovered, suggesting that constitutive expression of such pathway-specific activators represents a powerful approach for the discovery of novel bioactive natural products.

### The metabolism-remodeling approach

Although activation of biosynthetic genes at the transcriptional level is primarily important for exploiting useful metabolites, perturbation of biosynthesis by modulating, for example, the supply of precursors may also be a promising approach to enhancing the cell’s capability to produce secondary metabolites, a process termed metabolic engineering (Olano et al. 2008). One method of enhancing the yields of secondary metabolites consists of modulating fatty acid biosynthesis using small molecules. For example, screening of a large number (>30,000) of small molecules, seeking candidates that might “remodel” the yields of actinorhodin in *S*. *coelicolor*, identified 19 compounds that caused elevated or precocious production of actinorhodin (Craney et al. 2012). Further examination of four of these 19 molecules, antibiotic remodeling compounds (ARC)2, 3, 4, and 5, showed that (1) low concentrations of ARC2 enhanced the yield of actinorhodin, (2) the ARC2 effect can be observed in other actinomycetes, suggesting that the molecular target and mechanism of ARC2 is conserved in various actinomycetes, and (3) several of the most active molecules are structurally similar to the known antimicrobial agent triclosan, an inhibitor of fatty acid synthesis. The ARC2 effects therefore involve the inhibition of the enoyl reductase activity of FabI, an enzyme that catalyzes the final and rate-limiting step in fatty acid biosynthesis. Since fatty acid and polyketide synthesis share the precursors acetyl-CoA and malonyl-CoA, partial inhibition of fatty acid synthesis could recruit those acyl-CoAs preferentially to polyketide biosynthesis.

The ARC2 series of molecules not only enhanced the yields of known antibiotics, but induced the production of as yet unidentified compounds in *Streptomyces peuceticus*, compounds that could not be detected in the absence of ARC2 (Craney et al. 2012). Conceivably, the expression levels of the genes encoding the enzymes to synthesize these compounds are very low (i.e., nearly but not entirely silent), so that reinforcement of the biosynthetic process by an efficient supply of substrate made possible their detection. Alternatively, some other yet-unknown mechanism may be involved in the observed phenomenon. Theoretically, this approach may be enhanced by combination with the transcription-associated approaches (Ochi and Okamoto 2012). It may be possible to develop similar approaches for other classes of antibiotics, such as aminoglycosides and nonribosomal peptides, to access the full spectrum of secondary metabolites.

### Approach from cell-to-cell interactions

Co-culture is an effective method of inducing the production of cryptic metabolites. Although some co-culture methods have been reported, these methods are often specific to two bacterial strains. This limitation may be overcome by a novel fermentation method, the combined culture method, involving the co-culture of two bacterial strains (Onaka et al. 2011). Mycolic acid-containing bacteria could influence the biosynthesis of cryptic natural products in *Streptomyces* spp. The production of red pigment by *S*. *lividans* was induced by co-culture with *Tsukamurella pulmonis*, a mycolic acid-containing bacterium. Importantly, co-culture with *T*. *pulmonis* improved or inhibited natural product biosynthesis in 88 % of the *Streptomyces* strains isolated from soil: the production of new secondary metabolites was detected in 37 % of strains, while increased metabolite production was detected in 55 % of strains. The other mycolic acid-containing bacteria, *Rhodococcus erythropolis* and *Corynebacterium glutamicum*, improved or inhibited biosynthesis in 87 and 90 % of the *Streptomyces* strains, respectively: the production of new secondary metabolites was detected in 32 and 24 % of strains, respectively. Co-culture of *T*. *pulmonis* with *Streptomyces endus* led to the identification of a novel antibiotic, alchivemycin A. The addition of mycolic acid into the medium of pure *S*. *lividans* cultures had no effect on antibiotic production, suggesting that mycolic acid localized to the outer cell layer of the inducer bacterium affected secondary metabolism in *Streptomyces*, with this activity resulting from the direct cell-to-cell interaction of the two bacterial strains (Onaka et al. 2011). This method is also scalable for inducing the production of cryptic antibiotics, as it only involves the addition of a mycolic acid-containing bacterium to a pure culture of an actinomycete.

## Applicability of ribosome engineering to myxobacteria

Interest in myxobacteria, an important source of novel classes of secondary metabolites, has increased. The genomes of myxobacteria are large (9.14 Mb in *Myxococcus xanthus* and 13.03 Mb in *Sorangium cellulosum*), similar or larger than the genome of *S*. *coelicolor* (8.67 Mb). Myxobacterial genomes have been found to encode many genes involved in the synthesis of secondary metabolites (e.g., 8.6 % of the *M*. *xanthus* genome), opening the possibility of discovering clinically relevant natural products (Goldman et al. 2006; Schneiker et al. 2007; Wenzel and Muller 2009). Of the myxobacteria, the genus *Sorangium* is particularly valuable, as 46 % of metabolites isolated from myxobacteria are derived from this genus. Most myxobacterial metabolites are polyketides, nonribosomal peptides, and hybrids of the two structures. Hence, activation or enhancement of cryptic genes in the genus *Sorangium* is of interest, and the strategy of ribosome engineering may be utilized successfully for drug discovery.

## Applicability of ribosome engineering to eukaryotes

Fungi have been among the most important sources of biologically active secondary metabolites. Following the publication of the complete genome sequence of the model yeast *Saccharomyces cerevisiae* (Goffeau et al. 1996), hundreds of fungal genome projects have been carried out worldwide. As a result, large numbers of fungal genome sequences are now available publicly, including those of well-known producers of secondary metabolites, such as *Aspergillus oryzae* and *Penicillium chrysogenum* (Ma and Fedorova 2010). Their genomes, like those of *Streptomyces*, are also enriched in genes involved in secondary metabolite biosynthesis. Similar to *Streptomyces*, however, most biosynthetic gene clusters in fungi are either silent or expressed at very low levels under laboratory conditions. Thus, understanding the physiological conditions under which these genes are activated and developing a pragmatic approach for utilizing such genetic potential is important. A simple and yet efficient approach to isolate more secondary metabolites by exploring the genetic potential in fungi is to vary easily accessible cultivation parameters (Bode et al. 2002). Alternatively, physiological interaction among microorganisms may be practical. For example, co-cultivation of *Aspergillus nidulans* and *Streptomyces rapamycinicus* selectively stimulated cryptic fungal gene clusters involved in the biosynthesis of secondary metabolites, such as orsellinic acid, lecanoric acid, and the cathepsin K inhibitors F-9775A and F-9775B (Schroeckh et al. 2009). Small molecule epigenetic modifiers, such as the DNA methyltransferase inhibitor 5-azacytidine and the histone deacetylase inhibitor suberoylanilide hydroxamic acid, have been shown effective not only in altering secondary metabolite profiles but in generating new biomolecules (Williams et al. 2008). Likewise, nucleoid structure may be playing an analogous role to fungal chromatin structure in controlling transcriptional programs in actinomycetes, thus Moore et al. (2012) reported chemical elicitors that stimulate biosynthetic gene clusters in *Streptomyces*.

The concept based on bacterial ribosome engineering, as described above, has been applied to fungi to produce secondary metabolites. For example, introduction of gentamicin resistance into the marine-derived fungal strain *Penicillium purpurogenum* G59 effectively activated silent gene clusters for secondary metabolites (Chai et al. 2012). Although gentamicin did not inhibit this strain G59 during routine testing, treatment of G59 spores with high concentrations of gentamicin, in combination with dimethyl sulfoxide, inhibited strain growth, allowing the development of gentamicin-resistant colonies on agar plate. This method produced four antitumor secondary metabolites not found in the secondary metabolites of other *P*. *purpurogenum* strains. In addition, hygromycin B-resistant mutants of *Monascus pilosus* NBRC 4520 that exhibited enhanced production of secondary metabolites could be isolated using the general method used to obtain drug-resistant mutants in bacteria (Hosaka and Mizukami, unpublished data), because hygromycin B is an aminoglycoside antibiotic that potently inhibits protein synthesis in both prokaryotic and eukaryotic cells (Gonzalez et al. 1978). These findings indicate that modulation of ribosomal function may be applicable to a variety of fungi to elicit their potential secondary metabolism.

A new insight into the silent gene activation in the eukaryotic microorganisms is also coming from the ectopic ppGpp expression. The bacterial alarmone ppGpp, produced by RelA-SpoT homologue (RelA or RSH) on the ribosome in response to nutrient limitation, is a key signal factor to initiate the onset of bacterial secondary metabolism (Bibb 2005; Ochi 2007). Although *relA* and *RSH*, the genes encoding ppGpp synthetase, are distributed widely in bacteria and plants (Givens et al. 2004; Takahashi et al. 2004; Tozawa and Nomura 2011; van der Biezen et al. 2000), neither of these genes, nor ppGpp itself, has yet been identified in animals or eukaryotic microorganisms. Despite the essential lack of a *relA*-*spoT* homologue and ppGpp in the yeast *S. cerevisiae*, its heterologous expression of a *relA*-*spoT* homologue (*Sj*-*RSH*) isolated from the halophilic plant *Suaeda japonica* resulted in the accumulation of ppGpp, accompanied by the enhancement of tolerance against various stressors, including osmotic stress, ethanol, and freezing (Ochi et al. 2012; Yamada et al. 2003). Low levels of ppGpp [10–20 pmol (mg dry weight)^−1^] were sufficient for cellular stress tolerance without affecting growth rate (Ochi et al. 2012). These results raise an intriguing possibility that the ppGpp system can be applicable to the silent gene activation in such eukaryotic microorganisms as fungi.

## Applicability of rare earth elements to biotechnology

Despite the importance of rare earth elements (REEs) in the chemical industry, little is known about their biological effects in living cells. These elements, however, have recently been shown involved in the overproduction of antibiotics and in the activation of silent or poorly expressed genes in bacteria. The REEs consist of 17 elements, including scandium, yttrium, and the lanthanides (i.e., the 15 elements from lanthanum (La) to lutetium in the periodic table). Low concentrations (10–100 μM) of scandium (Sc) added to cultures of *S*. *coelicolor* (an actinorhodin producer), *Streptomyces antibioticus* (an actinomycin producer), and *S. griseus* (a streptomycin producer) were found to enhance antibiotic production 2–25-fold (Kawai et al. 2007). The effects of Sc were exerted at the level of transcription of pathway-specific positive regulatory genes, as demonstrated by marked upregulation of *act II*-ORF4 in *S*. *coelicolor*. Notably, REEs were effective in activating silent and poorly expressed secondary metabolite biosynthetic genes in *Streptomyces*. That is, the addition to the medium of low concentrations of Sc or La activated the expression of nine genes, 2.5 - to 12-fold, belonging to nine secondary metabolite–biosynthetic gene clusters of *S*. *coelicolor* (Tanaka et al. 2010). HPLC analysis of ethyl acetate-extractable metabolites indicated that several compounds could be detected only in the REE-treated cultures. This approach, due to its feasibility, should facilitate the discovery of new biologically active compounds. The ability of REEs (especially Sc) to enhance enzyme production and secondary metabolism was also observed in *B*. *subtilis*. The addition of Sc to the growth medium stimulated the production of both α-amylase and bacilysin at the transcriptional level (Inaoka and Ochi 2011).

REEs have long been known to have weak antimicrobial potency. Thus, it was possible to develop Sc-resistant mutants on plates containing Sc. As expected, actinorhodin overproducers (Ochi, unpublished results) and α-amylase overproducers (Inaoka and Ochi 2012) were often found among the mutants of *S*. *coelicolor* and *B*. *subtilis*, respectively. In *B*. *subtilis*, a mutation in the *uppS* gene, which encodes the enzyme undecaprenyl pyrophosphate synthase, was responsible for the Sc resistance phenotype and α-amylase overproduction. This *uppS86* mutation, however, did not affect the levels of *amyE* expression, suggesting that this mutation exerted its effects at the post-transcriptional level (Inaoka and Ochi 2012). Thus, the mechanism by which α-amylase production was stimulated by the *uppS86* mutation differed from that caused by the addition of Sc to the growth medium.

An important advantage of using REEs is that this method does not require any gene engineering technology or genomic information on the strains examined. Since REEs are distributed ubiquitously throughout the world, it is conceivable that microorganisms have acquired the ability to respond to low levels of these elements over the course of their long evolutionary history, possibly as a means of adapting their physiology to prevailing conditions. The effect of low concentrations of Sc on antibiotic production indicates that Sc functions in situ as a factor that induces or stimulates the production of secondary metabolites, including pigments, mycotoxins, phytotoxins, and antibiotics. “Rare earth microbiology” may thus offer new insight into entirely unknown regulatory events that occur in all organisms.

## Concluding remarks

The availability of genome sequence information on various microorganisms and newly developed methods of activating silent and poorly expressed genes suggest that natural product research has now entered a promising new era. Particular attention should be paid to combined approaches that include the transcription activation of key genes and metabolism remodeling. Reinforcement of biosynthetic processes by efficient substrate supply may be synergistic with transcription activation, eventually leading to the efficient discovery of new secondary metabolites. Another point of interest is the application of ribosome engineering to activate the silent biosynthetic pathways in fungi and myxobacteria, which may result in the efficient exploitation of novel secondary metabolites of these microbial groups. Also, since ppGpp enhances yeast cell tolerance to various stress stimuli, the ability of ectopic ppGpp in fungal cells to induce the synthesis of novel secondary metabolites is of interest. Apart from the technology, it would be important to envisage the reason why cryptic genes are silent in the laboratory fermentation conditions. Are the silent genes expressed under the special yet-unknown environmental conditions? If so, do the cryptic secondary metabolites play an intrinsic biological role(s) for producing organisms under the special environmental conditions? What are such special environmental conditions, if any? Understanding the mechanism(s) underlying the silencing of cryptic genes would help to fully untilize the microbial gene clusters for secondary metabolism.
